# Current News and Opinion

**Published:** 1885-05

**Authors:** 


### Current News and Opinion.

### OBITUARY.

#### J. G. AMBLER, M. D., M. D. S.

Died suddenly, at his residence in Dobbs Ferry, New York, on the evening of April 6th, of pneumonia, J. G. Ambler, M. D., M. D. S., in the sixty-ninth year of his age.

John Gardner Ambler was born in South New Berlin, Chenango County, New York, September 2, 1816. In the winter of 1832 he entered Rensselaer Polytechnic Institute, Troy, N. Y., and afterward studied medicine in Waterford, Saratoga County. He was a student of dentistry in the office of his uncle, Dr. D. C. Ambler, in New York City.

In 1836 he settled in Philadelphia, where he continued to reside until 1842, when he removed to New York City, and entered upon the practice of his profession. He at once gained a remunerative practice and in a few years became one of the leading dentists of New York City, practicing there until the day of his death.

Dr. Ambler always took an active interest in dental societies. He was one of the original members of the "Society of Dental Surgeons of the City of New York." He was a member of the American Dental Association, and took an especial interest in the American Dental Convention, having several times been elected President of that body, and by his energy prolonging its years of usefulness. In 1855 he edited and published the "Dental Monitor," a popular journal. During his professional career he contributed frequently to the literature of the profession.

Dr. Ambler was a communicant of the Protestant Episcopal Church, and took an interest in church work, for eighteen years being vestryman and warden of Christ Church, New York. His last literary effort was a eulogy upon the life and services of the late Rev. Geo. B. Reese, of Dobbs Ferry, N. Y., delivered before the congregation of Zion P. E. Church, of that place, on the evening of March 25th.

A. T.

"THE TOOTH CROWN PATENTS."

*Editor Independent Practitioner:*

Under the above title an article, signed by William H. Dwinelle, was published in the October (1884) number of your magazine, page 589.

That article purported to give an account of proceedings had on September 17, 1884, before Judge Wallace, in the United States Circuit Court for the Southern District of New York, in the suit of the International Tooth Crown Company vs. Berkowitz and Muller, and stated among other things that "the C. M. Richmond and Low Patents," owned by said Company, were at that time "invalidated," and that "the profession has nothing more to fear from them;" also, that "great confidence was felt that the Court would not enjoin parties under patents (meaning said patents) which it has decided to be invalid and concerning which the proof was positive that the party obtaining the patents and licenses under them knew that the patents were illegal when obtained, and that a great fraud was practiced upon the government and public by procuring, and seeking to enforce them."

False as we knew the statements to be concerning our patents, we decided to await the result of said Muller litigation before publishing a refutation of them.

We now announce to the profession,—

*First.* That not one of the fifteen patents owned by the International Tooth Crown Company has ever been "invalidated" or "decided to be void," either by His Honor Judge Wallace, or by any other judge or court, at any time or in any place.

*Second.* That certain technical objections having been raised to the issuing of injunction on the papers as originally prepared in the suit against Muller, the Company discontinued said suit and immediately sued Muller again for the same infringement on the same patents, and that in spite of the able defense interposed on his behalf, His Honor Judge Wallace has promptly enjoined said Muller from any further use of the Company's patents.

We may add that similar injunctions have been granted by the U. S. Circuit Court against Alvin S. Richmond and Cassius M. Richmond, and that the profession may understand that such injunctions are not to be trifled with, we call further attention to the fact that since the writing of said article by Dwinelle, Cassius M.

Richmond has, for disregard of such injunction, been sentenced to six months' imprisonment, to pay us a fine of one thousand dollars, and to stand committed after the expiration of said six months until we have received the amount of said fine; while Alvin S. Richmond has abandoned his practice here and departed for Europe since proceedings have been instituted against him for similar disregard.

It would thus appear that this company, instead of having any of its patents declared void, has proved overwhelmingly successful in all its attempts to enforce its legal rights, and that the profession would do well to post itself accurately as to the true facts concerning said patents and their validity.

Many of the profession are doubtless inclined to lend a ready ear to the libels and slanders constantly uttered against the company and its patents, for never in the history of the patent law has the public voluntarily submitted to payments of royalty, and the importance and value of a patent appears to be uniformly measured by the intensity of the resistance made to its enforcement.

The International Tooth Crown Company has thus far enforced and will continue to enforce as fast as possible, by legal means, the full penalty of the law against every dentist who steals its property or defames its reputation.

Time and patience only are needed for the full vindication of all the company's rights, and those practitioners are making a serious mistake who, relying on such articles as that of Dr. Dwinelle, fancy that they can disregard those rights with impunity.

Dated, New York, March 20, 1885.

International Tooth Crown Co.,
Lucius T. Sheffield,
Secretary and Treasurer.

---

CALIFORNIA.

Under the new dental law, registration under oath is compulsory, and a State Board of Examiners has been appointed, consisting of the following dentists: Morris J. Sullivan, Chas. W. Hibbard, Thos. Morffew and S. W. Dennis, of San Francisco; E. W. Biddle, Healdsburg; J. W. Hollinswell, Los Angeles; S. S. Southworth, Sacramento.

### GERMAN DENTAL FEES.

The legal fees for dental operations in the Kingdom of Prussia are not sufficient to insure an early competence for the practitioner. What would New York dentists say to the following fee bill, which is all that Prussian law allows the dentist.

| | |
|---|---|
| For extracting a tooth at the house of the dentist | 25 to 50 cents. |
| For extracting a root | 25 to 50 " |
| For extracting a number of roots, each | 19 to 25 " |
| For burring out a tooth | 37 to 50 " |
| For filling a tooth | 37 to 50 " |
| If a number of teeth are filled at once, for all after the first, one half price is paid. | |
| For boring into a tooth "to the nerve" | 37 to 50 cents. |
| For boring through a tooth to fasten artificial teeth to it | 37 to 50 " |
| Cleaning all the teeth | 75 to 2.25 |
| Filing off a sharp tooth | 25 to 50 cents. |
| Filing off a decayed tooth | 25 to 50 " |
| Filing between decayed teeth | 50 to 75 " |
| For first examination or consultation | 12 to 25 " |
| For each succeeding examination | 6 to 12 " |
| For regulating a tooth for a child | 37 to 50 " |
| For a number of teeth at once, each | 37 " |

A dentist in Leipzig lately sent a bill for seventy-five dollars (a very reasonable amount), but could only collect by law eight dollars.

---

### DENTAL SOCIETY OF THE STATE OF NEW YORK.

The above Society will meet at Albany, May 13th and 14th. The Board of Censors will meet at the same place Tuesday, May 12th, for the examination of candidates for the degree of M. D. S. For full information as to the requirements of the Board, application should be made to Dr. F. French, Rochester.

J. EDW. LINE,
ROCHESTER, April 15, 1885. Secretary.

---

### MASSACHUSETTS AND CONNECTICUT VALLEY DENTAL SOCIETIES.

A union meeting of the Massachusetts and Connecticut Valley Dental Societies will be held at Worcester, Mass., June 24 and 25, 1885. The combined membership of these two societies is a sufficient guarantee of a good meeting.

Per order EXECUTIVE COMMITTEE.

### DENTAL JOURNALS WANTED.

A fair price in cash will be paid for the following volumes and numbers of dental journals, or an exchange will be made with those desirous of completing their files. If dentists will look over their old journals and see what they have to spare, and communicate with the editor, a mutually profitable bargain may possibly be made.

*American Journal of Dental Science—Third Series.*

Vol. I, Nos. 2, 3, 4, 6, 7, 8, 9, 10, 11, 12.
Vol. II, Nos. 3, 4, 5, 6, 7, 8, 10.
Vol. III, Nos. 3, 4, 6, 7, 8, 9, 10, 11, 12.
Vol. IV, Nos. 1, 2, 3, 4, 5, 6, 7, 8.
Vol. V, Nos. 1, 2, 3, 4, 6, 7, 8, 9, 10, 11, 12.
Vol. VI, Nos. 1, 2, 3, 4, 5, 6, 7, 8, 9, 10, 11, 12.
Vol. VII, Nos. 1, 2, 3, 4, 6, 7, 8, 9, 10.
Vol. VIII, Nos. 1, 3, 4, 6, 7, 8, 10, 11, 12.
Vol. IX, No. 11.
Vol. XII, Nos. 4, 6, 11, 12.
Vol. XIII, Nos. 1, 2, 3, 4, 5, 6, 7, 8, 9, 10, 11, 12.
Vol. XV, Nos. 3, 6, 7, 8, 11, 12.

*Missouri Dental Journal.*

Vol. I, Nos. 2, 3, 5, 6, 9, 10.
Vol. IV, Nos. 3, 4, 5, 8, 12.
Vol. XI, Nos. 1, 3, 4, 5, 7, 8, 9, 10, 11, 12.

*Dental Register.*

Volumes II to XXII and XXXII to XXXVII.

*Items of Interest.*

Vol. II, Nos. 5, 6.

Vol. III, Nos. 8, 9.

*St. Louis Dental Quarterly.*

No. 3.

*Dental Luminary.*

Vol. I, Nos. 1, 2.

Vol. II, No. 4.

Vol. III, Nos. 1, 2.

Vol. IV, Nos. 1, 2, 3.

*Dental Headlight.*

Volumes I and II.

---

### ERRATA.

In the article by Dr. Clowes in the April No., page 191, third line from the bottom, read "pockets" for "sockets," and on page 192, fifth line from the top, read "rut" for "test," and the sense will be more complete.

### CHICAGO COLLEGE OF DENTAL SURGERY.

The third annual commencement exercises of the Chicago College of Dental Surgery took place at Hershey Music Hall, Chicago, Ill., on Friday evening, March 27, 1885, at 7.30 o'clock.

The address to the graduates was delivered by Prof. W. T. Belfield, M. D.; the valedictory by J. E. Hinkins, D. D. S.

The number of matriculants for the course of 1884-'85 was sixty-two.

The degree of D. D. S. was conferred on the following members of the senior class by Dr. J. A. Swasey, President of the Board of Directors:

H. Austin Armitage, M. D., England.
Harry Leon Barnum, M. D., Wisconsin.
Edward Everett Cady, Illinois.
Warren Cary, M. D., Illinois.
Jesse Austin Dunn, Illinois.
Astor Gerard Gray, Illinois.
Rudolph Theodore Hasselriis, Denmark.
Joseph Hickey, Dakota Territory.
John Edward Hinkins, Illinois.
A. Melville Hudson, Canada.
Charles Nelson Johnson, L. D. S., Ont.
William J. Johnson, M. D., Illinois.
Edmund Lambert, Illinois.
Asa Holt Lane, Illinois.
Charles William Lewis, Illinois.
Archibald Stewart McCandless, Ills.
Joseph Donahey Moody, Illinois.
Amos Jedd Nichols, Illinois.
Charles Putnam Pruyn, Illinois.
Joseph J. Reed, Illinois.
Charles Henry Wachter, Maryland.
George W. Whitefield, Illinois.

The honorary degree of D. D. S. was conferred upon Dr. E. B. Call, of Peoria, Ill.

TRUMAN W. BROPHY.
Secretary.

---

### CHICAGO DENTAL SOCIETY.

The annual meeting of the Chicago Dental Society was held on Tuesday evening, April 7th, and the following officers elected for the ensuing year:

*President*—Dr. C. F. Matteson.
*First Vice-President*—Dr. G. W. Nichols.
*Second Vice-President*—Dr. W. A. Stevens.
*Recording Secretary*—Dr. A. W. Hoyt.
*Corresponding Secretary*—Dr. P. J. Kester.
*Treasurer*—Dr. E. D. Swain.
*Librarian*—Dr. J. H. Wooley,

P. J. KESTER,
Corresponding Secretary.

### ANNUAL DINNER OF THE ALUMNI ASSOCIATION OF THE NEW YORK COLLEGE OF DENTISTRY.

The annual dinner for 1885 of the above-named Society, was partaken of at Delmonico's, on Saturday evening, March 7, 1885. President B. F. Luckey presiding.

The object for which this Association was organized, was to promote the best interests of the College, to keep up good fellowship among the graduates, and to advance professional interests. The organization has been very successful in its aims, and it is hoped will continue in a sphere of usefulness.

T. A. FLETCHER,
Chairman Executive Committee.

---

### NOTICE TO SUBSCRIBERS.

The treasurer of the INDEPENDENT PRACTITIONER would be pleased to receive remittances from subscribers who are in arrears. The most convenient method of remitting is to send P. O. notes.

Senders of P. O. money orders in Europe will confer a favor by notifying the treasurer by letter, at the same time that the orders are mailed.

If any subscriber has failed to receive back numbers due him, and will so inform the treasurer, efforts will be made to procure and forward the numbers designated.

C. E. FRANCIS,
33 West 47th Street, New York.

---

### A CORRECTION.

In the report of the fall meeting of the Connecticut Valley Dental Society, published in the *Archives of Dentistry,* I am made to say (intentionally or otherwise), that cocaine is a deadly poison. What I did say was, that *as many of the alkaloids were a deadly poison* it was best to exercise caution as to the strength used, until we had a better knowledge of the effect of this drug upon the system. The firm of whom I purchased my solution would only put up for my man a two per cent. preparation, leading me to infer that it was a matter of precaution, while in reality I discovered later that it was owing to the scarcity of the article.

EDWARD S. NILES.

BOSTON, April 1, 1885.

(53.) A case of arsenical poisoning came to me a few days since. Arsenical paste had been applied to a cavity in an inferior bicuspid, without being properly sealed up. The septum of bone between the tooth and the first molar has entirely sloughed away, and the sore does not heal. The outer and inner walls of the alveolus are evidently affected. I have used chloride of zinc and aromatic sulphuric acid with little effect. What else can I do? R.

(55.) Will somebody kindly inform me what produces, or what is the direct cause of the disease known as "Pyorrhœa Alveolaris?" G. B.

(54.) We find dentists using the terms "periodontitis," "peridentitis," "periostitis," and "pericementitis." They refer, of course, to the same disease. Now I would like to know which of these terms is most proper to use, or should be generally adopted? L. R.

(56.) The impression seems to prevail that chloroform is a more dangerous agent when administered as an anæsthetic, than ether. I would much like to know if this is a fact, and if so, how they differ in action on the system.
YOUNG DENTIST.

(57.) Will not some one who is an expert tell me how on earth the ordinary form of oxy-phosphate cements are used for capping exposed pulps? If it is mixed thin enough to flow without pressure, it will not "set" properly; while if it is so prepared as that it will set hard, it is too stiff to use without considerable pressure.

What can I do to make these cements wait a reasonable time before they get hard? The most of those that I have tried are quite as independent of any kind of manipulation as dried putty. JUNIUS.

### Answers.

LABORATORY. (43.) I say that all firm teeth remaining in the mouth should be retained, and only lost ones replaced with artificial teeth. The dentist who advises a clean sweep of natural teeth for the purpose of inserting artificial ones, ought to be cleanly swept from the profession. After using the substitutes given in return, miserable at best, thousands have had to deplore the loss of firm, natural teeth, that they had allowed to be extracted through such advice as "Laboratory" gives. He will do well never more to advise such a sacrifice, and his patients will be grateful to him for only restoring the lost, and helping them to save those remaining God-given members, which no art can equal. G. W. SPARROCK, D. D. S.,
Demerara, South America.

A. B. M. (51.) It may be interesting, in connection with other answers which you may receive in reply, for me to state that for the last three years, at least, I have invariably placed a small pellet of tin foil at the cervical margins of all proximal gold fillings, and also at other points along the margins of any cavity filled with gold, where for any pertinent reason decay was to be anticipated. I have yet to see a failure of such fillings from decay at those places. I do not attempt to explain the action of the tin, but reasoning from analogy I assume for tin so placed, about the same saving-power shown by tin *per se*, or by amalgam-patched gold fillings—a power which, so far as I am informed, has never been satisfactorily explained.

Some of our chemical friends may be able to enlighten us materially.
C. D. C.

We are always glad when correspondents sign their full name to their communications. Advice is worth more when one knows who is the adviser.—*Editor.*

